# Cost-effectiveness analysis of antiretroviral drugs for treatment-naive HIV infection in China

**DOI:** 10.1186/s12889-023-17052-1

**Published:** 2023-11-13

**Authors:** Min Li, Yuxin Cao, Hao Huang, Gang Qin, Minjie Chu, Meiyin Zou, Xun Zhuang

**Affiliations:** 1 Department of Epidemiology and Medical Statistics, School of Public Health, Nantong University, Chongchuan District, No.9 Seyuan Road, Nantong, Jiangsu China; 2 Zhangjiagang Center for Disease Control and Prevention, Department of Infectious Disease Prevention, No.18 Zhizhong Road, Zhangjiagang, Suzhou, Jiangsu China; 3 Nantong No.3 hospital affiliated to Nantong University, No.99 Qingnian Zhong Road, Chongchuan District, Nantong, Jiangsu China

**Keywords:** HIV, ART, Cost-effectiveness, Treatment naive, Markov model

## Abstract

**Introduction:**

Dolutegravir (DTG)-based regimen was included in the expanded formulary of China's National Free Antiretroviral Treatment Program at the end of 2021. Yet high price of DTG and lack of health economic evaluation in China present barriers for implementation of the regimen. The study aims to investigate the lifetime cost-effectiveness of DTG-based regimen for treatment-naive HIV infection in China.

**Methods:**

A decision-analytic Markov model was used to obtain the costs and effectiveness of four regimens: Arm A, efavirenz (EFV)-based regimen; Arm B, DTG-based regimen; Arm C, elvitegravir/cobicistat/tenofovir alafenamide/emtricitabine (EVG/c/FTC/TAF) regimen; Arm D, abacavir/lamivudine/dolutegravir (ABC/3TC/DTG) regimen. The potential impact of national centralized drug procurement policy was assessed in scenario analysis. The results were further validated through sensitivity analysis.

**Results:**

Compared with other three regimens, DTG-based regimen led to the fewest cumulative adverse reactions, opportunistic infections and deaths. Compared with EFV-based regimen, the base-case ICERs for DTG-based regimen were 13,357 (USD/QALY) and 13,424 (USD/QALY) from the healthcare system and societal perspective respectively. In the policy scenario analysis with the procurement price of DTG equal to that of LPV/r, DTG-based regimen would be dominant. The model results remained robust in sensitivity analyses.

**Conclusions:**

DTG-based regimen for treatment-naive patients is likely to be cost-effective and deserve wider implementation in China. This study strongly suggests the centralized procurement of DTG to minimize cost and maximize cost-effectiveness.

**Supplementary Information:**

The online version contains supplementary material available at 10.1186/s12889-023-17052-1.

## Background

The pilot for the China's National Free Antiretroviral Treatment Program (NFATP) began in Henan province in 2002, and the program fully began in 2003 [1]. Free first-line regimen for antiretroviral treatment (ART) is efavirenz (EFV) or nevirapine (NVP) + tenofovir disoproxil fumarate (TDF) or zidovudine (AZT) + lamivudine (3TC) [2]. This program has contributed greatly to case fatality rate reduction and life quality improvement [3]. However, there are ongoing challenges, including adverse reactions, drug resistance, and complications. A cohort study conducted on patients who had used free antiretroviral drugs for 4 or 5 years found that 69.9% of the patients had at least one adverse drug reaction [4].

In 2019, World Health Organization (WHO) [5] recommended dolutegravir (DTG, an integrase strand transfer inhibitor)-based regimens as the preferred first-line antiretroviral regimen for adults and adolescents because of the high potency and barrier to resistance, low incidence of adverse reactions, and virologic failure. DTG-based regimen was also the first-line antiretroviral regimen recommended by the United States and Europe [6] [7]. By the end of 2021, DTG was covered under the national free AIDS antiretroviral drug list in China [8]. This initiative not only provided an alternative for patients with severe central nervous system (CNS) symptoms and hepatotoxicity caused by EFV, but also ensured drug change for patients with multi-resistance of nucleoside and non-nucleoside reverse transcriptase inhibitors. However, DTG is still in the planning of centralized government procurement, and the average price per person-year for DTG is still as high as about USD 1 700 [9], far higher than the global price (USD 85 in lower-middle-income countries and USD 183 in upper-middle-income countries [10]), which may be the fundamental reason why DTG has not been really popularized in China (the utilization rate of DTG in first-line antiretroviral therapy is less than 3% in China). Considering the limited national budget for free drugs, economy is also an important criterion for drug selection in addition to drug effectiveness and safety. Although studies in Africa [11], France [12], India [13], Italy [14], Canada [15] and other countries have also confirmed that DTG was more cost-effective than EFV. The long-term economic value of DTG in the Chinese context has not been investigated previously.

The study aims to evaluate the lifetime cost-effectiveness of two first-line regimens (EFV-based regimen, DTG-based regimen) and two self-paying regimens for treatment-naive HIV infection from both health-care system and societal perspectives. Further, the study aims to explore the potential impact of national centralized drug procurement policy (NCDP) on the regimen selection.

## Methods

### Data sources

Details of costs and utilities, and clinical parameters were collected based on clinical data (from a designated AIDS hospitals in Jiangsu province [Nantong No.3 hospital affiliated to Nantong University], from November 2015 to November 2020, *N* = 2,934), government documents, published literature and expert opinions (four experienced clinical experts and several HIV-infected patients were interviewed). This study followed the recommendations of the Consolidated Health Economic Evaluation Reporting Standards (CHEERS) reporting guideline [16]. The study was approved by the Medical Ethics Committee of Nantong University (Approval number: No. [2020] 4). All participants signed an informed consent form.

### Model structure

**Fig. 1:**
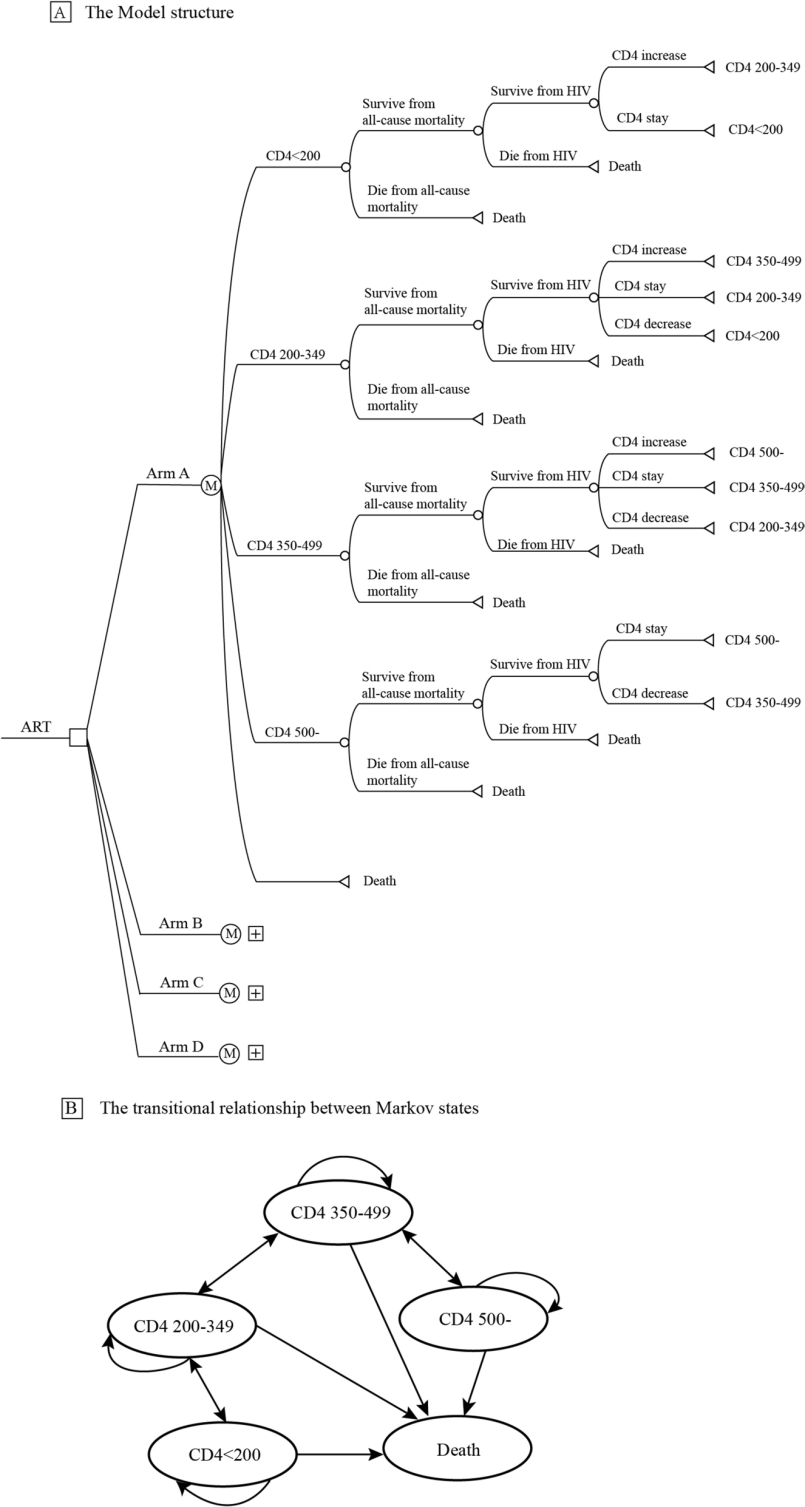
**A** The Model structure. **B** The transitional relationship between Markov states

A decision-analytic Markov model was built in this study, which consisted of a decision tree (Fig. 1A) reflecting the 4 regimens: Arm A: the current first-line free regimens (EFV-based regimen [EFV + TDF or AZT + 3TC], converted to LPV/r-based regimen [LPV/r + AZT or TDF + 3TC] in the fifth year); Arm B: DTG-based regimen (DTG + TDF + 3TC, has been incorporated into the national free AIDS antiretroviral drug list, to be implemented); Arm C: EVG/c/FTC/TAF (Genvoya, self-paying); Arm D: ABC/3TC/DTG (Triumeq, self-paying).

In China in 2020, there were about 81,000 newly-diagnosed HIV-infected individuals [17], among whom 92% initiated first-line ART during the same year [18]. Thus, it was assumed that the age at the start of the ART was 30 years old [19].Therefore, the cohort was set with 75,000 patients at the age of 30 years who initiated antiretroviral treatment (ART) following diagnosis of HIV infection per year. Due to the chronic condition of HIV/AIDS, a lifetime horizon of 40 years was applied, assuming a total lifetime expectancy of 70 years (life expectancy among people with HIV receiving ART were within 10 years of those of the general population [20], which is 76 years in China [21]). The Markov model consisted of four CD4 states (< 200, 200–349, 350–499, and 500- cells/μL) and one absorbing mortality state. The initial CD4 distribution were set as < 200: 30%, 200–349: 30%, 350–499: 20%, and 500-: 20% [22]. It was assumed that the CD4 states can only maintain the original state, transfer to the adjacent state, or directly lead to death in one cycle (one year), according to the related studies results that the CD4 cell counts of patients who receive ART can increase by approximately 150 cells/μL every year on average [23]. Figure 1B shows the transitional relationship between Markov states. TreeAge Pro 2021 (TreeAge Software, LLC, Williamstown, Massachusetts, USA) was used to build the model, and the cycle period was defined as one year with half cycle correction.

The main model outcomes included HIV-related health outcomes; total costs; QALYs; and incremental cost-effectiveness ratios (ICERs). Costs and ICERs were reported in 2020 US dollars. According to the WHO standard [24], cost-effectiveness was determined based on whether ICER was smaller than three times China’s GDP per capita (USD 31 241 in 2020) [25].

### Model inputs

From the societal perspective, the following cost items were considered in the model: (1) Direct medical costs, including ARV, testing, treatment of adverse reactions, and opportunistic infections; (2) direct non-medical costs, including transportation, accompanying care, and nutrition; (3) and indirect costs, including work stoppage. The human capital approach (HCA) [26] was used to calculate indirect costs, as follows [26]: indirect cost = wage standard × loss of working time. The wage standard was the per capita disposable income of national residents in 2020 [25]. The loss of working time was the average days of hospital stay per year, which were obtained from the hospital clinical data. All costs were converted to the values in 2020 using the Chinese consumer price index (CPI) [25], and were converted to US dollars at an exchange rate of 6.8974 Chinese yuan per dollar [21]. Further details of the costs are provided in the Table 1. The health-care system perspective only included the direct medical costs (eTable 1 in the Supplementary Data).

**Table 1:** Costs used in the model (USD per year, adjust the value to 2020)

Parameters	Value (range)^a^	Distribution	Sources
Direct medical costs			
ARV			
TDF (300 mg)	17.40 (± 25%)	Gamma	Government documents [9]
AZT (300 mg) (× 2)	52.12 (± 25%)		
3TC (300 mg)	16.44 (± 25%)		
EFV (600 mg)	126.29 (± 25%)		
LPV/r (200 mg/50 mg) (× 2)	509.68 (± 25%)		
DTG (50 mg)	1,704.72 (± 25%)		
EVG/c/ TAF / FTC (200 mg/10 mg/150 mg/150 mg)	2,243.97 (± 25%)		
ABC/3TC/DTG (600 mg/300 mg/50 mg)	5,009.78 (± 25%)		
Testing			
Testing before ART	61.32 (± 25%)	Gamma	Ma L et al., 2016 [27]
HLA-B*5701	101.47 (± 25%)		Clinical data combined with expert opinions
CD4 testing	74.51 (± 25%)		
Viral load	130.46 (± 25%)		
Routine testing (the first year)	144.96 (± 25%)		Ma L et al., 2016 [27]
Routine testing (following years)	72.48 (± 25%)		
Treating of the adverse reactions			
CNS symptoms	263.25 (± 25%)	Gamma	Guo Z et al., 2008 [28]
Digestive system diseases	503.44 (± 25%)		
Anemia	81.47 (± 25%)		
Hepatotoxicity	614.63 (± 25%)		
Dermatitis	401.68 (± 25%)		
Treating of the opportunistic infections			
Pneumocystis pneumonia	2,754.22 (± 25%)	Gamma	Guo Z et al., 2008 [28]
Cytomegalovirus infection	2,246.87 (± 25%)		
Tuberculosis	1,465.10 (± 25%)		
Herpes	633.76 (± 25%)		
Direct non-medical costs			
Transportation			
CD4 < 200	126.98 (± 25%)	Gamma	Ma L et al., 2016 [27]
CD4 200–349	151.05 (± 25%)		
CD4 350–499	178.01 (± 25%)		
CD4 500-	202.36 (± 25%)		
Accompanying care			
CD4: < 200	294.85 (± 25%)	Gamma	Ma L et al., 2016 [27]
CD4: 200–349	266.87 (± 25%)		
CD4: 350–499	57.40 (± 25%)		
CD4: 500-	52.19 (± 25%)		
Nutrition	186.56 (± 25%)	Gamma	expert opinions
Indirect costs (work stoppage)			
CD4 < 200	146.41 (± 25%)	Gamma	Clinical data
CD4 200–349	122.06 (± 25%)		
CD4 350–499	97.70 (± 25%)		
CD4 500-	85.38 (± 25%)		

Abbreviations: *ART* Antiretroviral therapy, *CD4* Cluster of differentiation 4, *USD* US dollar, *ARV* Antiretroviral drug, *TDF* Tenofovir disoproxil fumarate, *AZT* Zidovudine, *3TC* Lamivudine, *EFV* Efavirenz, *LPV/r* Lopinavir/ritonavir, *DTG* Dolutegravir, *EVG/c/ TAF / FTC* Elvitegravir/cobicistat/tenofovir alafenamide/emtricitabine, *ABC/3TC/DTG* Abacavir/lamivudine/ dolutegravir, HLA-B*5701 Human leukocyte antigen-B*5701, *CNS* Central nervous system

^a^The range was the maximum and minimum of all literature estimates, or ± 25%

QALY was used as the utility measurement. The utility values (Table 2) were associated with four CD4 states, adverse reactions, and opportunistic infections. Costs and utility values were analyzed at a discount rate of 5% per annum [29].

**Table 2:** Utilities used in the model

Parameters	Value (range)^a^	Distribution	Sources
Utilities (QALY)			
CD4 < 200	0.781 (0.603–0.863)	Beta	Tsevat et al., 1999 [30] combined with expert opinions
CD4 200–349	0.833 (0.719–0.933)		
CD4 350–499	0.868 (0.784–0.970)		
CD4 500-	0.888 (0.798–0.970)		
Adverse reactions			
CNS symptoms	UD-0.033 (± 25%)	Beta	Kauf TL et al., 2008 [31]
Digestive system diseases	UD-0.009 (± 25%)		
Anemia	UD-0.013 (± 25%)		
Hepatotoxicity	UD-0.012 (± 25%)		
Dermatitis	UD-0.010 (± 25%)		
Opportunistic infections			
Pneumocystis pneumonia	UD-0.15 (± 25%)	Beta	Simpson KN et al., 2013 [32]
Cytomegalovirus infection	UD-0.15 (± 25%)		
Tuberculosis	UD-0.15 (± 25%)		
Herpes	UD-0.15 (± 25%)		

Abbreviations: *QALY* Quality-adjusted life-year, *CD4* Cluster of differentiation 4, *CNS* Central nervous system, *UD* Utility decrement

^a^: The range was the maximum and minimum of all literature estimates, or ± 25%

The transition between different states was reflected by the transition probability, including the change rate of CD4 count status and mortality (Table 3). The change rate of CD4 count status were estimated by clinical experts according to the virological inhibition rate and the patient’s immunological reestablishment [33]. The cumulative transition probability was converted into the annual transition rate using the formula [34] $r=[-\mathrm{log}\left(1-prob\right)]/t$ (r, annual incidence rate; prob, cumulative transition probability for t years). The instantaneous incidence rate for each event was converted into the annual transition probability included in the model using the formula [35] $p=1-\mathrm{exp}(-r*t)$ (p, annual transition probability; r, annual incidence rate; t, time,). Other parameters that reflected the effects of regimens included incidences of adverse reactions, and opportunistic infections (eTable2 in the Supplement Data).

**Table 3:** Population and transition probabilities used in the model

Parameters	Value (range)^a^	Distribution	Sources
Age at the start of the ART (year)	30 (20–40)	Normal	Dou Z et al., 2015 [19]
Switching time of arm A (year)	5 (2–10)	Normal	Clinical data combined with expert opinions
Initial CD4 distribution			
CD4 < 200	30 (± 25%)	Beta	Clinical data combined with expert opinions
CD4 200–349	30 (± 25%)		
CD4 350–499	20 (± 25%)		
CD4 500-	20 (± 25%)		
The Transitional Probabilities			
CD4 increase (%)			
Arm A before switching	61.13 (44.25–63.00)	Beta	Cohen C et al., 2012 [36]
Arm A after switching	52.50 (45.00–54.75)		Aboud M et al., 2019 [37]
Arm B	69.75 (55.88–70.50)		Sax PE et al., 2017 [38]
Arm C	69.38 (66.30–69.75)		Sax PE et al., 2015[39]
Arm D	66.00 (64.50–69.75)		Walmsley SL et al., 2013 [40]
CD4 maintain (%)			
Arm A before switching	22.23 (18.85–22.60)	Beta	Cohen C et al., 2012 [36]
Arm A after switching	20.50 (19.00–20.95)		Aboud M et al., 2019 [37]
Arm B	23.95 (21.18–24.10)		Sax PE et al., 2017 [38]
Arm C	23.88 (23.26–23.95)		Sax PE et al., 2015 [39]
Arm D	23.20 (22.90–23.95)		Walmsley SL et al., 2013 [40]
HIV mortality (%)			
CD4 < 200	8.0 (± 25%)	Beta	Lewden C et al., 2007 [41]
CD4 200–349	1.8 (± 25%)		
CD4 350–499	1.2 (± 25%)		
CD4 500-	0.7 (± 25%)		
All-cause mortality by age	China population and employment statistics yearbook	Beta	China population and employment statistics yearbook, 2020 [42]
Discount rate (%)	0–8	^−^	Liu G et al., 2020 [29]

Abbreviations: *ART* Antiretroviral therapy, *CD4* Cluster of differentiation 4, *HIV* Human immunodeficiency virus

^a^The range was the maximum and minimum of all literature estimates, or ± 25%

### Scenario and sensitivity analyses

In 2021, Chinese government issued the National Centralized Drug Procurement (NCDP) policy for drug price and cost control [8]. We conducted a policy scenario assuming the procurement price of DTG equal to that of LPV/r. The stability of the model was evaluated by one-way sensitivity analysis and probabilistic sensitivity analysis (PSA) for all parameters. The ranges of parameters for one-way sensitivity analysis were based on maximum and minimum values reported in the literature, when available. For unavailable ranges, the values of ± 25% were adopted. Using a second-order Monte Carlo simulation (5,000 iterations), PSA was performed to examine the effects of all parameters’ distributions. Further information on the parameter range and distribution is provided in Tables 1, 2 and 3.

## Results

### Clinical outcomes

Based on the simulated cohort of 75,000 HIV infection, over lifetime horizon, 30,790 cases of death occurred in arm B (DTG-based regimen), reducing 9,729 cases than arm A (the free regimen); 82,734 cases of adverse reactions occurred in arm B, reducing 546,678 cases than arm A; 50,677 cases of opportunistic infections occurred in arm B, reducing 31,254 cases than arm A. The HIV-related health outcomes of Arm C (Genvoya) and arm D (Triumeq) were inferior to that of arm B, with more cases of deaths, adverse reactions, and opportunistic infections (Table 4).

**Table 4:** HIV-related health outcomes of four arms (simulated cohort of 75,000)

	**Arm A**	**Arm B**	**Arm C**	**Arm D**
Cumulative cases of each state				
CD4 < 200	2,176 (2,087–2,268)	28 (19–40)	34 (24–48)	146 (132–172)
CD4 200–349	4,503 (4,376–4,632)	321 (287–358)	368 (331–407)	932 (873–993)
CD4 350–499	9,300 (9,124–9,479)	3,622 (3,508–3,739)	3,856 (3,738- 3,976)	5,844 (5,701–5,990)
CD4 500-	18,502 (18,271–18,735)	40,239 (39,971–40,507)	39,838 (39,570–40,106)	35,923 (35,654–36,192)
Death	40,519 (40,251–40,787)	30,790 (30,526–31,055)	30,905 (30,641–31,170)	32,156(31,890–32,422)
Death averted	-	9,729 (9,549–9,911)	9,614 (9,435–9,795)	8,363 (8,195–8,534)
Cumulative cases of HIV-related diseases				
Adverse reactions	629,412 (627,423–631,377)	82,734 (81,055–84,428)	236,461 (233,966–238,964)	179,392 (177,103–181,692)
Adverse reactions averted	-	546,678 (544,282–549,064)	392,951 (390,264–395,635)	450,020 (447,383–452,652)
Opportunistic infections	81,931 (80,262–83,621)	50,677 (50,425–50,928)	51,168 (50,917–51,418)	56,366 (56,133–56,598)
Opportunistic infections averted	-	31,254 (30,989–31,519)	30,763 (30,499–31,028)	25,565 (25,311–25,820)

Abbreviations: *CD4* Cluster of differentiation 4, *HIV* Human immunodeficiency virus

### Cost and cost-effectiveness outcomes

Pairwise incremental analysis (cost and effectiveness comparisons between two arms) were conducted based on the China Guidelines for Pharmacoeconomic Evaluations (2020) [29], from the health-care system perspective. First, all arms were ranked according to their costs, from low to high, that is, USD 1,181,508,179, USD 2,384,721,209, USD 3,003,817,442, and USD 6,024,007,347 for arms A, B, C, and D, respectively. Second, an incremental analysis was conducted between arms C and D because of their higher costs; arm D costs more than arm C (6,024,007,347 > 3,003,817,442) but with fewer QALYs (954,888 < 968,102). As arm D was a more strictly dominated regimen, it was eliminated first. Subsequently, arms C and B were compared; arm C cost more than arm B (3,003,817,442 > 2,384,721,209), but with fewer QALYs (968,102 < 972,746); therefore, arm C was eliminated. Finally, for the remaining arms A and B, arm B increased total costs by USD 1,203,213,030 and QALYs by 972,746, with an ICER of 13,357 (USD/QALY), which was smaller than three times GDP per capita of USD 31,241, indicating that the increased cost of arm B was worthwhile. Thus, arm B (the last regimen retained from all incremental analyses) was the most cost-effective regimen (see Table 5). In terms of cost-effectiveness, arm C (Genvoya) and arm D (Triumeq) were generally inferior to arm B.

**Table 5:** Results for base-case and scenario analysis (simulated cohort of 75,000)

	**Total costs, USD**	**Incremental costs, USD**	**Effectiveness, QALY**	**Incremental effectiveness, QALY**	**ICER relative to status quo**	**ICER relative to next best strategy**
Health-care system perspective						
Base case						
Arm A	1,181,508,179	-	882,663	-	-	-
Arm B	2,384,721,209	1,203,213,030	972,746	90,083	13,357 (vs arm A)	13,357 (vs arm A)
Arm C	3,003,817,442	1,822,309,263	968,102	85,439	21,329 (vs arm A)	Dominated by arm B
Arm D	6,024,007,347	4,842,499,168	954,888	72,225	67,047 (vs arm A)	Dominated by arm B
Policy scenario						
Arm A	1,181,508,179	-	882,663	-	-	Dominated by arm B
Arm B	1,052,060,583	-129,447,596	972,746	90,083	Dominant	Dominant
Arm D	1,104,777,298	-76,730,881	954,888	72,225	Dominant	Dominated by arm B
Arm C	1,109,497,655	-72,010,524	968,102	85,439	Dominant	Dominated by arm B
Societal perspective						
Base case						
Arm A	1,796,066,692	-	882,663	-	-	-
Arm B	3,005,359,690	1,209,292,998	972,746	90,083	13,424 (vs arm A)	13,424 (vs arm A)
Arm C	3,624,234,388	1,828,167,696	968,102	85,439	21,397 (vs arm A)	Dominated by arm B
Arm D	6,642,498,920	4,846,432,228	954,888	72,225	67,102 (vs arm A)	Dominated by arm B
Policy scenario						
Arm A	1,796,066,692	-	882,663	-	-	Dominated by arm B
Arm B	1,672,699,064	-123,367,628	972,746	90,083	Dominant	Dominant
Arm D	1,723,268,871	-72,797,821	954,888	72,225	Dominant	Dominated by arm B
Arm C	1,729,914,601	-66,152,091	968,102	85,439	Dominant	Dominated by arm B

Abbreviations: *USD* US dollar, *QALY* Quality-adjusted life-year, *ICER* Incremental cost-effectiveness ratio

Similarly, from the societal perspective, arm D and arm C were strictly dominated regimen and were eliminated from the ranking in turn. Arm B increased total costs by USD 1,209,292,998 and QALYs by 90,083, with an ICER of 13,424 (USD/QALY). Arm B was the most cost-effective regimen.

### Scenario and sensitivity analyses

In a scenario analysis assuming three self-paying regimens were included in the national free AIDS antiretroviral drug list, at a price on parity with LPV/r, it significantly reduced the total costs of arms B, C, and D. Arm B resulted in a dominant regimen, with lower costs and greater QALYs than all other arms from the health-care system and societal perspectives (Table 5).

**Fig. 2:**
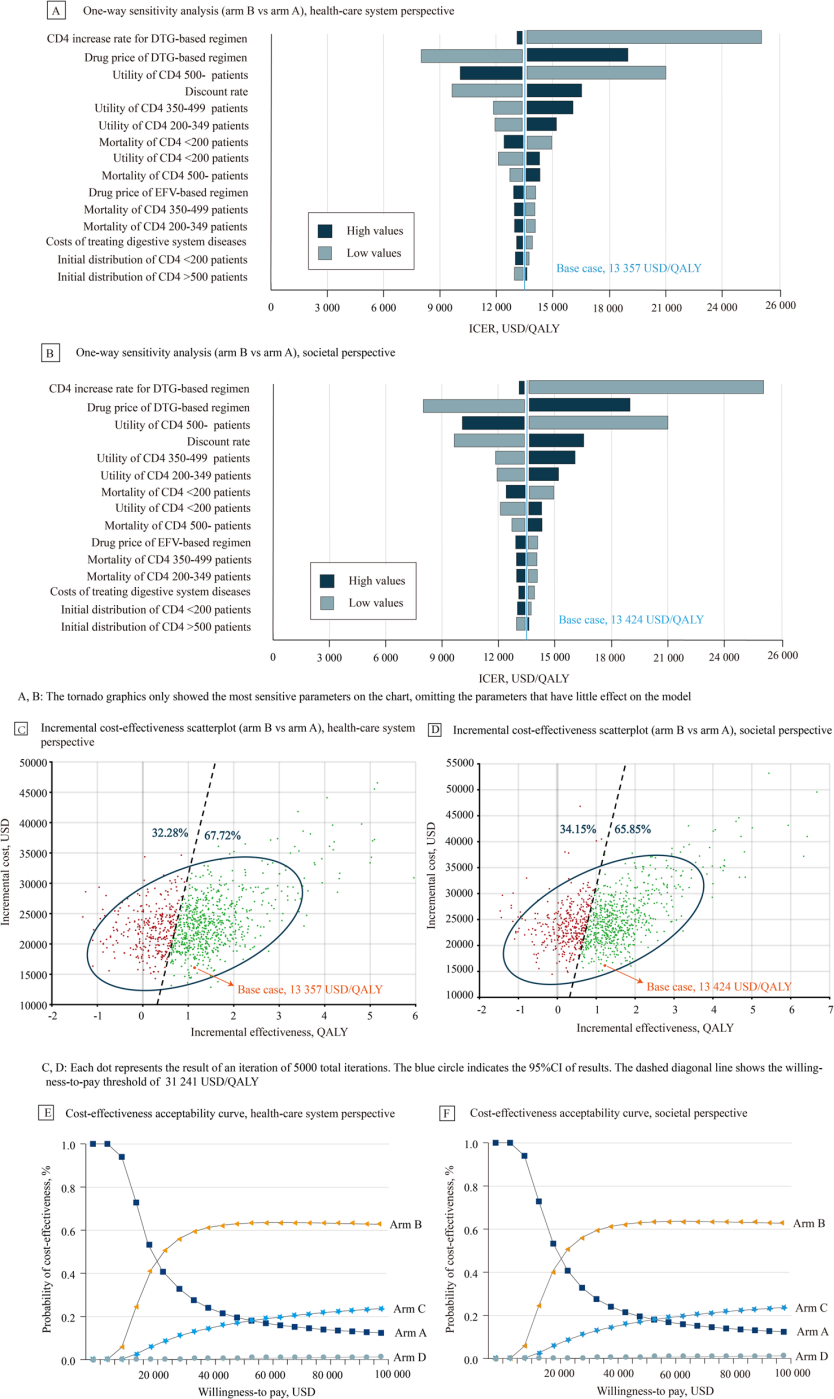
**A** One-way sensitivity analysis (arm B vs arm A), health-care system perspective. **B** One-way sensitivity analysis (arm B vs arm A), societal perspective. **C** Incremental cost-effectiveness scatterplot (arm B vs arm A), health-care system perspective. **D** Incremental cost-effectiveness scatterplot (arm B vs arm A), societal perspective. **E** Cost-effectiveness acceptability curve, health-care system perspective. **F** Cost-effectiveness acceptability curve, societal perspective

The model results remained robust in sensitivity analyses. From health-care system and societal perspective, one-way sensitivity analysis suggested that the variables that had a significant association with ICER (arm A vs arm B) were the CD4 increase rate for arm B, drug price of arm B, and utility of the CD4 > 500 patients (Fig. 2A, B). Notably, all ICERs were less than three times China’s GDP per capita (USD 31,241) during one-way sensitivity analysis. In PSA, after a second-order Monte Carlo simulation (5,000 iterations), the cost-effectiveness acceptability curve (Fig. 2E, F) showed the probability of the four arms being cost-effective in a range of willingness-to-pay (WTP). Considering a WTP of three times of China’s GDP per capita (USD 31,241), arm B had 58.11% probability of being cost-effective among four arms from the health-care system perspective (Fig. 2E), and 56.81% probability of being cost-effective among four arms from the societal perspective (Fig. 2F). Moreover, an incremental cost-effectiveness scatterplot (Fig. 2C, D) of arm B vs arm A was conducted, which found that there was 67.72% probability that arm B was cost-effective versus arm A at a WTP USD 31,241 from the health-care system perspective (Fig. 2C), and 65.85% probability from the societal perspective (Fig. 2D). Therefore, we could consider arm B to be the most cost-effective option. One-way sensitivity analysis and incremental cost-effectiveness scatterplot of arm B vs arm C and arm B vs arm D were shown in eFigure1-eFigure 2 (in the Supplementary Data).

## Discussion

Although DTG has been included in the national AIDS free drug list at the end of 2021, it still takes some time and effort to implement the national centralized procurement. The average price per person-year for DTG is still as high as about USD 1,700 [9], far higher than the global price (USD 85 in lower-middle-income countries and USD 183 in upper-middle-income countries [10]), which may be the fundamental reason why DTG has not been really popularized in China (the proportion of DTG in first-line antiretroviral therapy is less than 3% in China). The study aims to evaluate the lifetime cost-effectiveness of DTG-based regimen for treatment naive HIV infection from societal and health-care system perspective, and to simulate the influence of price change on cost-effectiveness after the update of the list. The study aims to evaluate the lifetime cost-effectiveness of DTG-based regimen for treatment naive HIV infection from societal and health-care system perspective, and to simulate the influence of price change on cost-effectiveness after the implementation of centralized procurement.

In this study, a decision-analytic Markov model was developed to compare the lifetime cost-effectiveness of the existing free regimens (EFV-based regimen, converted to LPV/r-based regimen in the fifth year) and three self-paying regimens (DTG-based regimen, Genvoya, and Triumeq) for treatment-naive HIV infection, from a societal perspective. The results showed that DTG-based regimen significantly reduced the cases of deaths, adverse reactions, and opportunistic infections. From the health-care system and societal perspective, DTG-based regimen resulted in ICERs of 13,357 (USD/QALY) and 13,424 (USD/QALY) compared with the free regimen, respectively. In a scenario analysis assuming three self-paying regimens were simulated in the national free AIDS antiretroviral drug list at the price of parity to LPV/r, DTG-based regimen was a dominant regimen, with lower costs and greater QALYs than all comparators. These findings provided an economic argument supporting the 2019 WHO guidelines and the recently updated national free AIDS antiretroviral drug list.

To our knowledge, only one study [43] had performed a 5-year cost-effectiveness analysis of DTG-based regimen in China, compared to EFV-based regimen and LPV/r-based regimen, using a Markov model. It reported that DTG dominated (with fewer costs and higher QALYs) in both settings, when DTG was priced on parity to LPV/r. However, the long-term economic value of DTG in the Chinese context has not been investigated previously. Our study evaluated the cost-effectiveness of self-paying regimens at the current market price and considered lifetime horizon.

It was found that patients who used EFV-based regimen were more likely to switch to LPV/r-based regimen because of severe adverse drug reactions and drug resistance when collecting data from research field hospitals, which was consistent with the findings in the literature [44–46], and the average time of switching change was five years [47]. Therefore, in this study, arm A was set as initial treatment with EFV-based regimen and converted to LPV/r-based regimen in the fifth year (second-tenth year in sensitivity analysis). Arm A was the most used in Chinese clinical settings, with a market share of nearly 90%. Arm B (DTG-based regimen) was the preferred first-line antiretroviral regimen recommended by the WHO [5], and was covered under the national free AIDS antiretroviral drug list in China by the end of 2021[8]. Arm C (Genvoya) was included in China’s reimbursement list in 2019 [48], and arm D (Triumpq) was recommended by the guidelines of the United States [6] and the European Union [7].

From the societal perspective, direct medical costs, non-direct medical costs, and indirect costs were included in this study. The health-care system perspective only included the direct medical costs. HIV management costs were included in the medical service costs (such as testing, treatment of adverse reactions, and opportunistic infections). Due to the difference in major types of adverse reactions among regimens, for example, EFV-based regimen was CNS symptoms, while LPV/r-based regimen was digestive system diseases. The major adverse reactions of each regimen (CNS symptoms, digestive system diseases, anemia, hepatotoxicity, and dermatitis) were included as much as possible. In addition, ABC in Arm D may cause hypersensitivity, once it occurs, the drug should be stopped for lifetime [33]. On the other hand, the clinical experts believe that Arm D is a compound preparation of ABC/3TC/DTG, with a big size tablet, which led to the decrease the tolerance and compliance of patients, thus reducing the treatment effects. This may be the reasons for the difference in health outcomes of arms B and D despite their similar elements.

When collecting data from research field hospitals, it was found that most of the patients who used self-paying regimens were advanced treatment-naive or drug-resistant patients, which was consistent with the consensus of Chinese clinical experts [49]. Therefore, the efficacy of self-paying regimens might be underestimated, and the costs of treating adverse reactions might be overestimated. Moreover, when calculating the indirect costs, hospital stay was assessed as work stoppage hours, ignoring non-hospital hours, such as productivity loss due to drug-induced CNS symptoms. This might underestimate the indirect costs, especially for the EFV-based regimen. For these reasons, the cost-effectiveness of the DTG -based regimen may be underrated.

Our study has some strengths. First, this is the first study to estimate the costs and health effects of antiretroviral regimen in the long term in the Chinese context. Second, it considered more comprehensive regimens, including four major ART regimens in the Chinese market, which accounted for more than 90% of the market share [50]. In addition, actual drug prices in the domestic market were adopted in this study, and the clinical parameters were combined with hospital data, reflecting the real status quo in China. The study found that DTG-based regimen for treatment-naive patients is likely to be cost-effective. The reduction in infections can also be attributed to the immediate decrease in viral load resulting from the implementation of the DTG-based regimen, which highlights the need for broader adoption in China. The findings of this study may serve as a scientific foundation for the development of national strategies for AIDS prevention and control, as well as for the rational allocation of resources.

This study’s limitations are related to the representativeness of the model parameters. First, due to the lack of large-sample efficacy data for DTG, some clinical parameters were obtained from small-sample RCTs, which might be different from long-term real-world data. Second, QALY was estimated using the utility derived from overseas literature, as these data were not available on the Chinese literature. We will adjust further the data if there are future reports of utility values in China. Third, in PSA, a certain proportion in the incremental cost-effectiveness scatterplot had near-zero effectiveness, which may render ICERs ineffective as a tool for assessment. Finally, as this study neither measured treatment-experienced patients nor considered rising trend of HIV infection in China, the impact of DTG-based regimen may under-estimated.

## Conclusions

DTG-based regimen for treatment-naive patients is likely to be cost-effective and deserve wider implementation in China. This study strongly suggests the centralized procurement of DTG to minimize cost and maximize cost-effectiveness.

### Supplementary Information

**Additional file 1: eTable 1. **Cost-Effectiveness Impact Inventory demonstrating types of health outcomes and costs included in each perspective. **eTable 2. **Annual incidences of adverse reactions and opportunistic infections. **eFigure 1.** One-way Sensitivity Analysis (arm B vs arm C, arm B vs arm D). **eFigure 2. **Incremental Cost-effectiveness Scatterplot (arm B vs arm C, arm B vs arm D).

## Data Availability

The dataset supporting the conclusion of this article is available upon reasonable request from the corresponding author.

## Abbreviations

3TC: Lamivudine
ABC/3TC/DTG: Abacavir/lamivudine/dolutegravir
ART: Antiretroviral treatment
AZT: Zidovudine
CHEERS: Consolidated Health Economic Evaluation Reporting Standards
CNS: Central nervous system
CPI: Chinese consumer price index
DTG: Dolutegravir
EFV: Efavirenz
EVG/c/FTC/TAF: Elvitegravir/cobicistat/tenofovir alafenamide/emtricitabine
HCA: Human capital approach
ICER: Incremental cost-effectiveness ratio
NCDP: National Centralized Drug Procurement policy
NFATP: National Free Antiretroviral Treatment Program
NVP: Nevirapine
PSA: Probabilistic sensitivity analysis
QALYs: Quality-adjusted life years
TDF: Tenofovir disoproxil fumarate
WHO: World Health Organization
WTP: Willingness-to-pay
