# Supplementary material for: Cost-effectiveness analysis of antiretroviral drugs for treatment-naive HIV infection in China
Source: BMC Public Health. 2023 Nov 13;23:2228. doi: 10.1186/s12889-023-17052-1 (PMC10641994; doi:10.1186/s12889-023-17052-1)
Supplement: Supplementary file 1 — Additional file 1: eTable 1. Cost-Effectiveness Impact Inventory demonstrating types of health outcomes and costs included in each perspective. eTable 2. Annual incidences of adverse reactions and opportunistic infections. eFigure 1. One-way Sensitivity Analysis (arm B vs arm C, arm B vs arm D). eFigure 2. Incremental Cost-effectiveness Scatterplot (arm B vs arm C, arm B vs arm D). [file 12889_2023_17052_MOESM1_ESM.docx]

**eTable 1.** **Cost-Effectiveness Impact Inventory demonstrating types of health outcomes and costs included in each perspective**

| **Sector** | **Type of impact** | **Included in analysis from the**  **following perspective?** | |
| --- | --- | --- | --- |
|  |  | **Health-care system** | **Societal** |
| Health outcomes | Adverse reactions | 🗸 | 🗸 |
|  | Opportunistic infections | 🗸 | 🗸 |
|  | Death | 🗸 | 🗸 |
| Direct medical costs | ARV | 🗸 | 🗸 |
|  | Testing | 🗸 | 🗸 |
|  | Treatment of adverse reactions | 🗸 | 🗸 |
|  | Treatment of opportunistic infections | 🗸 | 🗸 |
| Direct non-medical costs | Transportation | 🗸 | 🗴 |
|  | Accompanying care | 🗸 | 🗴 |
|  | Nutrition | 🗸 | 🗴 |
| Indirect costs | Work stoppage | 🗸 | 🗴 |

**eTable 2. Annual incidences of adverse reactions and opportunistic infections**

| **Parameters** | **CNS symptoms** | **Digestive diseases** | | **Anemia** | **Hepatotoxicity** | | **Dermatitis** | | **Range** | **Distribution** | **Sources** |
| --- | --- | --- | --- | --- | --- | --- | --- | --- | --- | --- | --- |
| **Annual incidences of adverse reactions** | | | | | | | | | | | |
| Arm A before switching | 21.25 | 6.19 | | 6.50 | 6.91 | | 7.12 | | ±25% | Beta | ENCORE1 trial [1]  Behrens G et al, 2014 [2]  Mendes JC et al, 2018 [3] |
| Arm A after switching | 5.16 | 33.87 | | 4.19 | 0.00 | | 3.23 | | ±25% | Beta | DAWNING trial [4] |
| Arm B | 1.72 | 4.35 | | 0.07 | 0.00 | | 1.28 | | ±25% | Beta | SPRING-2 trial [5] |
| Arm C | 8.60 | 8.70 | | 0.08 | 0.00 | | 3.85 | | ±25% | Beta | GS-US-292-0111 trial [6] |
| Arm D | 5.88 | 8.59 | | 0.00 | 0.00 | | 1.82 | | ±25% | Beta | SINGLE trial [7] |
| Parameters | Pneumocystis pneumonia | | Cytomegalovirus infection | | | Tuberculosis | | Herpes | | Distribution | Sources |
| **Annual incidences of opportunistic infections** | | | | | | | | | | | |
| CD4 <200 | 17.44  （0.89-44.6） | | 16.15  （1.96-31.71） | | | 2.73  （0.88-8.84） | | 5.82  （3.06-9.23） | | Beta | Freedberg KA et al, 2007 [8]  Kimmel AD et al, 2005 [9]  Ye W et al, 2020 [10]  You C et al, 2021 [11] |
| CD4 200- | 2.83  （0.42-11.11） | | 5.66  （0.11-11.11） | | | 0.30  （0.26-3.70） | | 2.60  （0.00-3.70） | | Beta | Freedberg KA et al, 2007 [8]  Kimmel AD et al, 2005 [9]  Ye W et al, 2020 [10]  You C et al, 2020 [11] |
| CD4 350- | 0.44  （0.00-3.33） | | 2.64  （0.03-10.00） | | | 0.00  （0.00-0.07） | | 2.60  （1.93-3.33） | | Beta | Freedberg KA et al, 2007 [8]  Kimmel AD et al, 2005 [9]  Ye W et al, 2020 [10] |
| CD4 500- | 0.10  （0.00-0.21） | | 1.72  （0.03-7.69） | | | 0.00  （0.00-0.03） | | 1.07  （0.00-2.13） | | Beta | Freedberg KA et al, 2007 [8]  Kimmel AD et al, 2005 [9]  Ye W et al, 2020 [10] |

Abbreviations: CNS, central nervous system


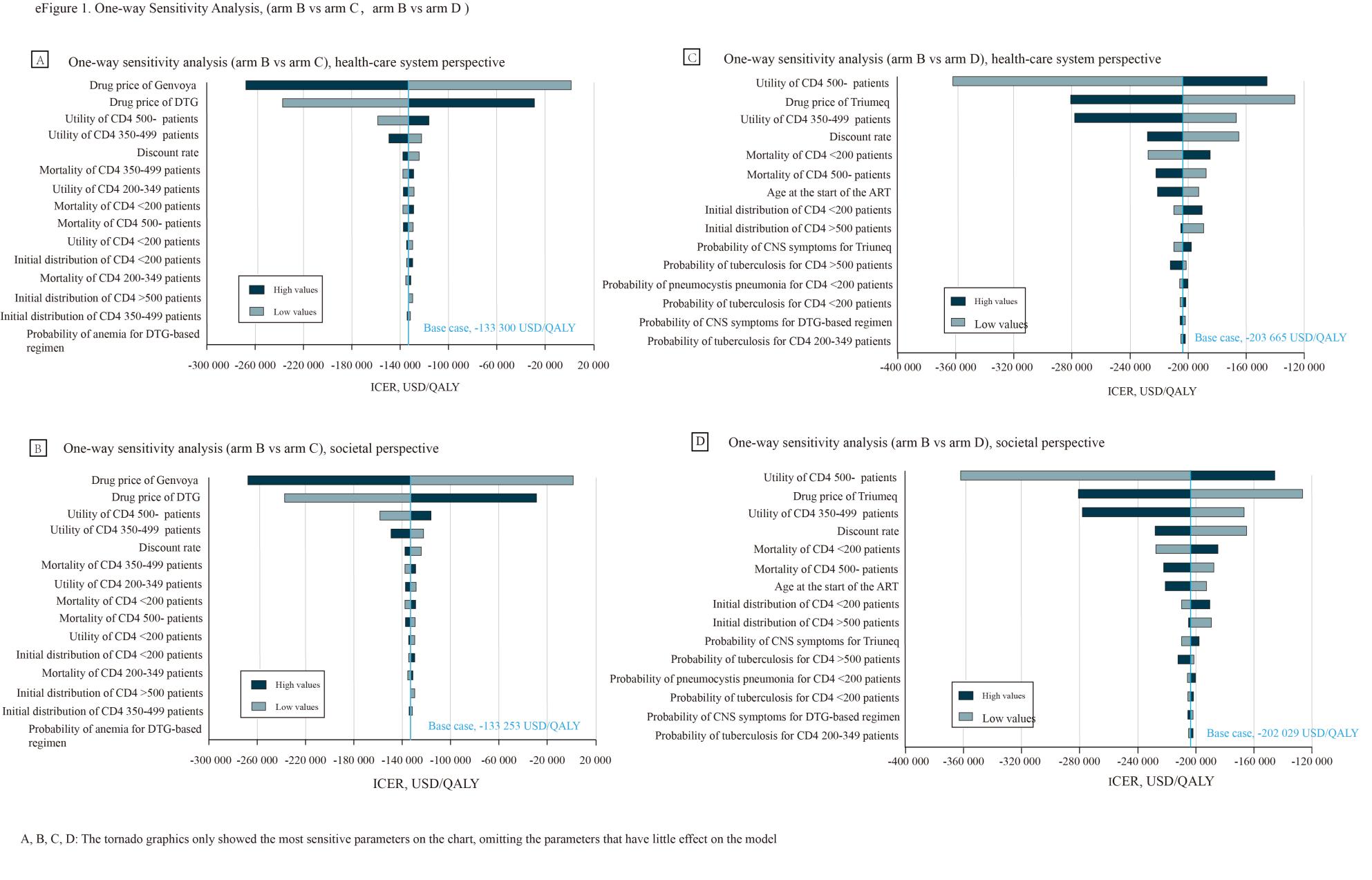


eFigure 1. One-way Sensitivity Analysis (arm B vs arm C, arm B vs arm D)


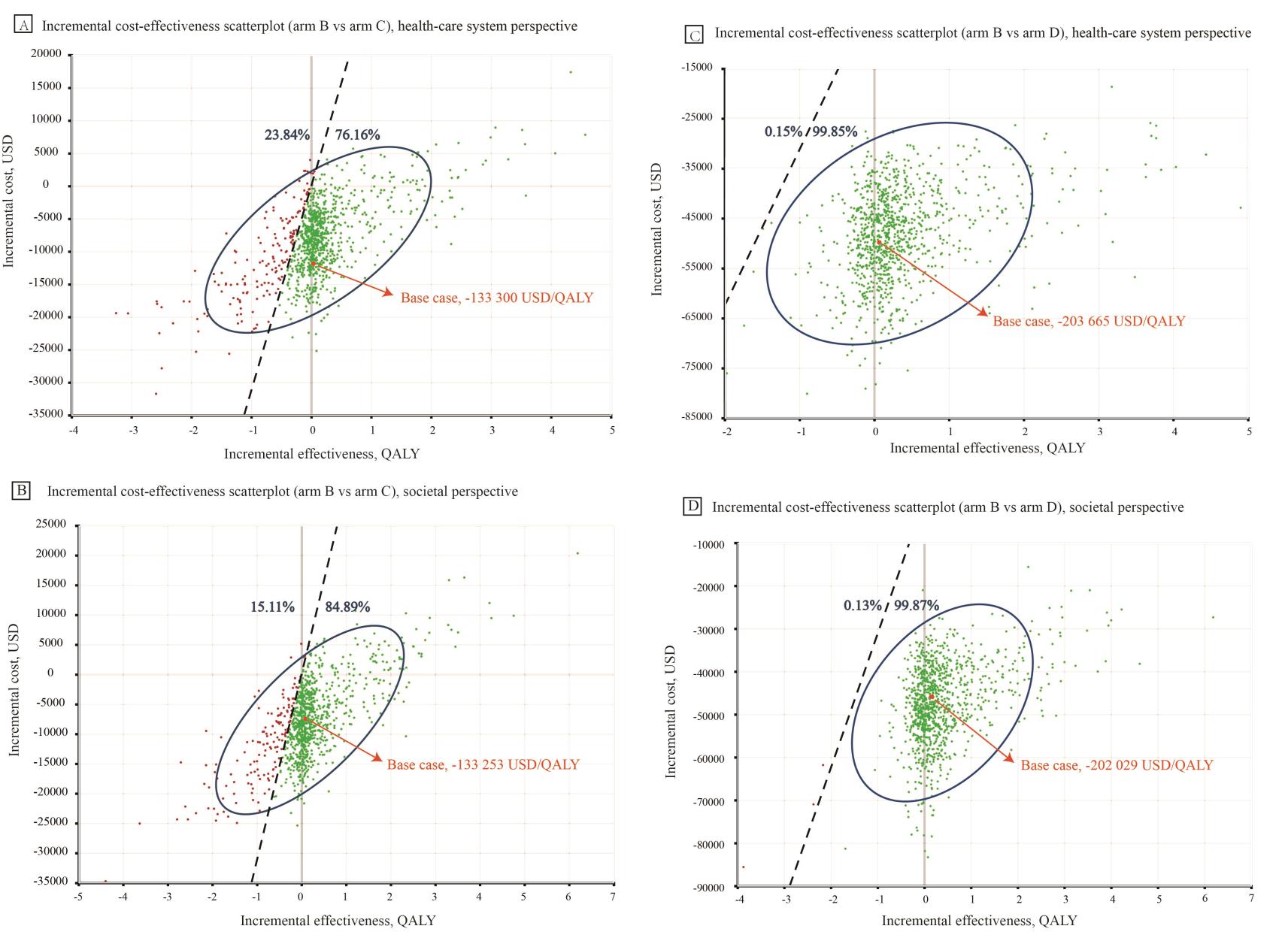


eFigure 2. Incremental Cost-effectiveness Scatterplot (arm B vs arm C, arm B vs arm D)

**Extended Reference**

1. Safety and efficacy of reduced versus standard dose efavirenz (EFV) plus two nucleotides in antiretroviral-naïve adults. (ENCORE1). <https://clinicaltrials.gov/ct2/show/results/NCT01011413?term=NCT01011413&draw=2&rank=1>. Published 21 Feb 2020/Accessed 6 Aug 2021.
2. Behrens G, Rijnders B, Nelson M, et al. Rilpivirine versus efavirenz with emtricitabine/tenofovir disoproxil fumarate in treatment-naïve HIV-1-infected patients with HIV-1 RNA ≤100,000 copies/mL: week 96 pooled ECHO/THRIVE subanalysis. AIDS Patient Care STDS. 2014 Apr;28(4):168-75. doi:10.1089/apc.2013.0310
3. Mendes JC, Bonolo PF, Ceccato MDGB, et al. Adverse reactions associated with first-line regimens in patient initiating antiretroviral therapy. Eur J Clin Pharmacol. 2018 Aug;74(8):1077-1088. doi:10.1007/s00228-018-2472-y
4. Comparative efficacy and safety study of dolutegravir and lopinavir/ritonavir in second-line treatment. <https://clinicaltrials.gov/ct2/show/results/NCT02227238?term=NCT02227238&draw=2&rank=1>. Published 1 Apr 2021/Accessed 6 Aug 2021.
5. A trial comparing GSK1349572 50mg once daily to raltegravir 400mg twice daily (SPRING-2). <https://clinicaltrials.gov/ct2/show/results/NCT01227824?term=NCT01227824&draw=2&rank=1>. Published 9 Oct 2018/Accessed 6 Aug 2021.
6. Study to evaluate the safety and efficacy of E/C/F/TAF versus E/C/F/TDF in HIV-1 positive, antiretroviral treatment-naive adults. <https://clinicaltrials.gov/ct2/show/results/NCT01797445?term=NCT01797445&draw=2&rank=1>. Published 2 Mar 2020/Accessed 6 Aug 2021.
7. A trial comparing GSK1349572 50mg plus abacavir/lamivudine once daily to atripla (also called the SINGLE trial). <https://clinicaltrials.gov/ct2/show/results/NCT01263015?term=NCT01263015&draw=2&rank=1>. Published 4 Apr 2018/Accessed 6 Aug 2021.
8. Freedberg KA, Kumarasamy N, Losina E, et al. Clinical impact and cost-effectiveness of antiretroviral therapy in India: starting criteria and second-line therapy. AIDS. 2007 Jul;21 Suppl 4(Suppl 4):S117-28. doi:10.1097/01.aids.0000279714.60935.a2
9. Kimmel AD, Goldie SJ, Walensky RP, et al. Optimal frequency of CD4 cell count and HIV RNA monitoring prior to initiation of antiretroviral therapy in HIV-infected patients. Antivir Ther. 2005;10(1):41-52. doi:10.1016/j.antiviral.2004.10.002
10. Ye W, Wu S, Chen Y, et al. Clinical characteristics of HIV/AIDS patients with opportunistic infection different baseline levels of CD4^+^T cells receiving antireviral therapy. Chinese Journal of Nosocomiology. 2020 Jul;30(15):2296-2300. doi:10.11816/cn.ni.2020-192317
11. You C, Tang J, Zhou L, et al. Analysis of opportunistic infections in patients with AIDS at different CD4 levels. Clinical Medicine. 2021 Jan;41(01):1-3. doi:10.19528/j.issn.1003-3548.2021.01.001
